# Clinical effects of different virtual reality presentation content on anxiety and pain: a randomized controlled trial

**DOI:** 10.1038/s41598-023-47764-8

**Published:** 2023-11-22

**Authors:** Yoshio Yamashita, Reona Aijima, Atsushi Danjo

**Affiliations:** Department of Oral and Maxillofacial Surgery, Faculty of Medicine, Saga University, Nabeshima 5-1-1, Saga, 849-8501 Japan

**Keywords:** Medical research, Therapeutics

## Abstract

Many patients are frightened of or anxious about dental treatment. We have recently reported our use of virtual reality (VR) to alleviate the fear and anxiety experienced during oral surgical procedures However, the effectiveness of VR in alleviating anxiety varies greatly between individuals. We therefore investigated whether the content of the VR presentation made any difference to its effect in alleviating anxiety, and whether it had any analgesic effect. The study subjects experienced one of two different types of VR presentation and were asked to complete a questionnaire about any changes in their anxiety during the procedure, including a visual analog scale (VAS) score. As an objective evaluation, changes in pain threshold during the VR presentation were investigated using PainVision. For those patients who experienced a presentation showing a natural landscape, the change in VAS score was − 13.3 ± 28.7 mm, whereas for those who experienced a presentation showing a video game the change was − 22.2 ± 32.1 mm, an even greater reduction. In a pain questionnaire completed by individuals who had experienced the video game presentations, approximately 70% reported that their pain had diminished. An objective evaluation of pain threshold also showed that the pain threshold of individuals increased by around 3% while experiencing the natural landscape VR presentation, but that while experiencing the video game presentation, it increased significantly by around 15% compared with baseline. These results show that the content of the presentation affected not only the rate of decrease in anxiety, but also the pain threshold.

## Introduction

During oral surgery, patients are required to keep their mouths open for a certain period, and the metallic screech of the surgical drill and the unpleasant smell generated by the drilling of hard tissue such as teeth and bone all contribute to a high level of stress^1,2^. In particular, the face is usually covered with a cloth to keep operations hygienic and protect the eyes, obscuring the patient’s view, and this generates high levels of anxiety and fear. Even when local infiltration anesthesia is highly effective, it can be difficult to relieve the patient’s anxiety and fear. Conventionally, intravenous or inhalation anesthesia, or oral tranquilizers have been used for patients with severe anxiety or fear, such as those with dental phobia, but there are limits to the use of drugs from the perspectives of both the patient and the institution, and they are not suitable for use in every case. Preoperative anxiety is also reportedly associated with complications, problematic behavior, and emotional distress^3^. Methods of sedation should ideally involve as little physical invasiveness and burden as possible. Recent studies have shown that using aromatherapy^4^ or listening to specific music^5^ during dental treatment is effective in alleviating anxiety. We have recently reported that the use of virtual reality (VR) is subjectively and objectively effective in alleviating the fear and anxiety experienced during impacted mandibular third molar extraction^6^ or other minor oral surgical procedures^7^ conducted under local anesthesia.

VR refers to the techniques and systems used to create an environment that is not itself a physical original, but functions essentially in the same way by stimulating perception, including the user’s five senses. A major advantage of VR is that because it involves almost no physical invasion, potential drug complications or adverse drug events, it may provide a safe method of sedation^8^. Our previous study also showed that it is highly effective in alleviating anxiety even for older patients, and since it can be used by a wide range of age groups, it has the potential to become a highly versatile tool.

However, problems have also surfaced. The VR experiences that we prepared and presented to patients featured landscapes and animals that were hardly moving, with the aim of inducing relaxation^6,7^. However, some patients who experienced them, particularly younger men, complained that this content was “boring” or “uninteresting,” and for them it failed to produce the desired anxiolytic effect. This suggested that the response to the nature of the presentation might vary depending on factors such as the patient’s preferences, personality, and sex, and that the content of the VR presentation might thus modulate its effectiveness. In this study, patients were therefore asked to experience different presentations, and whether these resulted in any differences in the degree to which their anxiety was diminished was investigated. We evaluated whether experiencing a newly created presentation with content that required thought and concentration, separately from the previously used presentation of natural landscapes, would change their decrease in anxiety. Whether experiencing different presentations would make any difference to the pain threshold was also examined.

## Results

Table 1 shows the compositions of the three randomly assigned groups. There was no significant difference among the groups in age and gender. The completion rate of the surgical procedure on patients fitted with VR was 100%, and the extraction was not discontinued because of VR use in any case. No patient complained of motion-sickness-like symptoms after experiencing VR.

**Figure 1:**
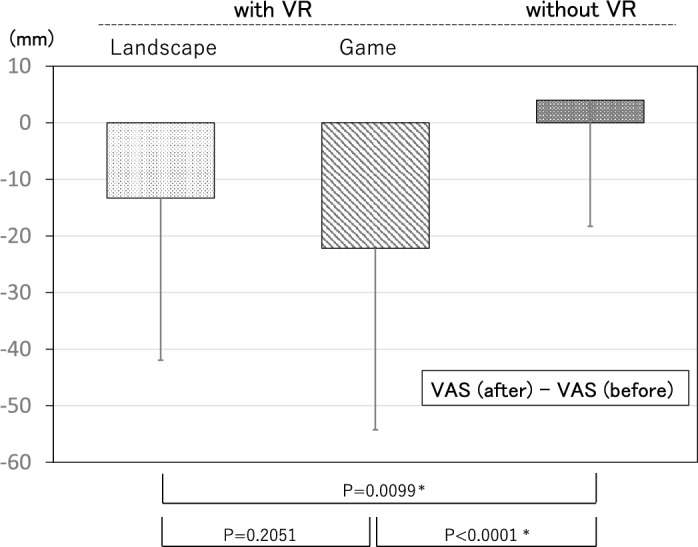
Changes in VAS scores for anxiety. Difference between the preoperative VAS score for anxiety and that during the procedure. Anxiety improved among patients who used VR, whereas it increased in patients who did not use VR. Error bars represent mean ± SD.

**Figure 2:**
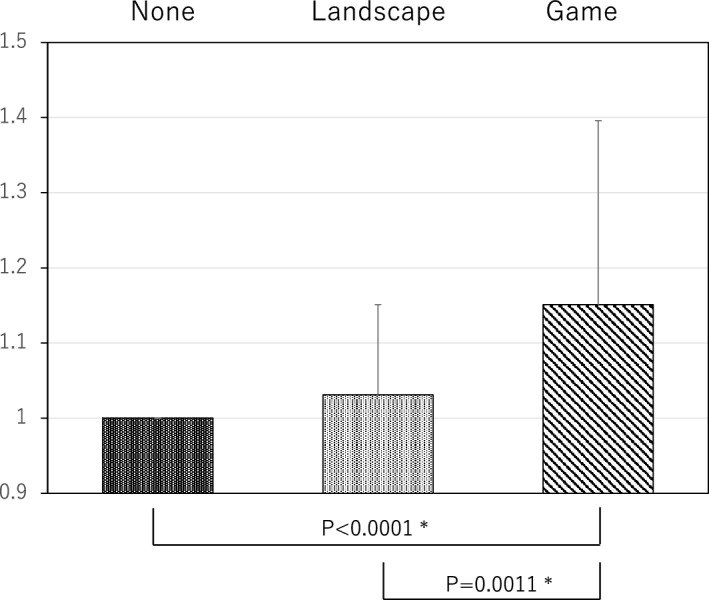
Changes in pain threshold. Variations in electric current measured by PainVision. The current is compared with the baseline value while the subject was not using VR (none). Error bars represent mean ± SD.

1. The results of the satisfaction questionnaire are illustrated in Table 2. No patient in either group responded that it was Unacceptable. In both groups, the reason for choosing the Poor response was that the images were not in focus during the presentation.
2. A comparison of the results of the decreased anxiety questionnaire between the natural landscape VR and video game VR groups showed high rates of decrease in both groups, with 80% of those in the natural landscape VR group and 84% of those in the video game VR group responding that it had either Decreased Substantially or Decreased (Table 3).
3. An investigation of differences between the preoperative and postoperative VAS scores for anxiety and fear found that, as in our previous report, in the non-VR group, the VAS score for anxiety tended to increase postoperatively by 4.0 ± 22.3 mm compared with that preoperatively. In the two VR groups, however, the VAS score decreased significantly compared with the score for the non-VR group. A comparison between the two VR presentations showed that, for those patients who experienced a presentation showing a natural landscape (*n* = 51), the decrease in VAS score was − 13.3 ± 28.7 mm, whereas for those who experienced a presentation showing a video game (*n* = 73), the decrease was − 22.2 ± 32.1 mm, an even greater reduction (Fig. 1). However, there was no clearly significant difference between the natural landscape VR and video game groups.
4. The results of the pain questionnaire by the patients who experienced the video game presentation were Decreased Substantially 21%, Decreased 47%, Unchanged 32%, Increased Slightly 0%, and Increased 0%, with approximately 70% reporting that their pain had diminished (Table 4).
5. In the investigation of changes in pain threshold using PainVision, the percentage change in current while experiencing the natural landscape VR and the video game VR compared with the value without VR as baseline was calculated. In terms of the mean threshold shift, the threshold increased by around 3% compared with baseline while experiencing the natural landscape presentation. However, while experiencing the video game presentation, it increased significantly by approximately 15% compared with baseline (Fig. 2).

**Table 1:** Patient demographics.

	VR (+)		VR (−)
	Landscape	Game	
Gender (%)			
Female	24 (47.0)	47 (64.4)	35 (72.9)
Male	27 (53.0)	26 (35.6)	13 (27.1)
Age (ys)			
Mean	27.6 ± 7.7	29.4 ± 9.5	34.9 ± 11.8
Range	20–47	20–57	20–65

Data bare represented as a mean ± standard deviation of the mean. No significant differences were found between three groups.

**Table 2:** Response to the satisfaction questionnaire by VR use.

	Excellent (%)	Good (%)	Fair (%)	Poor (%)	Unacceptable (%)
Landscape (n = 51)	34	48	12	6	0
Game (n = 73)	42	34	20	4	0

**Table 3:** Response to the questionnaire on the alleviation of fear and anxiety by VR use.

	Greatly decreased (%)	Decreased (%)	Unchanged (%)	Slightly increased (%)	Increased (%)
Landscape (n = 51)	28	52	20	0	0
Game (n = 73)	33	51	16	0	0

**Table 4:** Response to the questionnaire on the alleviation of pain during treatment by VR use.

	Greatly decreased (%)	Decreased (%)	Unchanged (%)	Slightly increased (%)	Increased (%)
	21	47	32	0	0
(n = 73)	68

## Discussion

Many patients are frightened of or anxious about dental treatment^9^. One major reason is that the treatment site is in the facial area itself, so that the treatment can be seen and remembered. Pain and fear patients have felt during previous dental treatment frequently become imprinted, and many patients feel stressed prior to treatment. In wisdom tooth extraction, even if the patient’s vision is obscured, they are required to keep their mouths open for a certain period, and the metallic screech of the surgical drill and the unpleasant smell during drilling all contribute to a high level of stress. In practice, even if adequate analgesia is provided by local anesthesia, patients still experience mental and physical stress during the procedure. In some cases, this may even lead to dental phobia. The methods used to relieve this anxiety and fear of treatment include sedation by inhalation nitrous oxide/oxygen anesthesia or intravenous sedation/anesthesia, as well as the use of oral tranquilizers. However, drug administration may cause side effects or be contraindicated, and is thus not suitable for all patients.

In a previous study, we demonstrated that the use of VR decreased anxiety and fear of surgical treatment under local anesthesia both subjectively and objectively^6,7^. In our objective assessment using heart rate variability (HRV)^10,11^, we showed that, although patients were in a state of arousal preoperatively, with sympathetic nervous system activity predominating, experiencing VR with images designed to induce relaxation restored balance with parasympathetic nervous activity, and they became relaxed.

VR is currently in use in several medical fields^12–14^. It consists of three elements: three-dimensional presence, autonomy, and real-time interaction. It can thus transport the user into a different experience by stimulating all the senses, not just sight^15^. The employment of VR techniques such as the one used in this study does not require the use of specific drugs and involves almost no physical invasion. A further advantage is that, because it requires neither drugs nor expensive equipment, it does not entail any running costs. Our previous results also demonstrated that it can be used by a wide range of age groups, including older people. The results of the present study showed that the content of the VR presentation modulated its effect on decreasing anxiety. It was also demonstrated, both subjectively and objectively, that a video game presentation that required thought and concentration reduced pain. Another study has reported that the use of VR during burn treatment enables the dose of analgesics to be reduced, a finding that supports our results^16–18^. Although there is individual variation in its effects on reducing anxiety and pain, the effects appear to be dependent on the individual’s level of concentration while experiencing the presentation. This suggests that the viewer’s preferences may be a major factor in the selection of a presentation. The physiological mechanism that diminishes pain during concentration on a presentation has yet to be clearly identified. We intend to use near-infrared spectroscopy (NIRS) or dynamic magnetic resonance imaging (MRI) to measure changes in cerebral perfusion to demonstrate the extent to which these effects can be obtained in future^19,20^.

One point that warrants attention in the experience of VR is cybersickness^21–23^. This condition is known to occur when the viewer’s gaze follows text or images moving rapidly on the screen, which results in headaches, nausea, and feelings of unsteadiness. There are calls for particular care to be taken with children^24,25^. In this study, even the video game presentation that we produced did not cause any patient to complain of cybersickness-like symptoms. As the age range expands, however, care will be required in the future to check that cybersickness does not occur while experiencing VR.

A head-mounted display is required to experience VR, but the thickness and heaviness of these displays is an impediment for both operator and patient. It is hoped that smaller, lighter devices will be developed in the future.

In a previous study, we showed that most patients who used VR during a surgical procedure expressed the wish to use VR during other such procedures in the future^6,7^. This suggested that VR is readily accepted by patients and can fill a need as an anxiolytic device. The recent universal adoption of smartphones and the development of the games industry have increased the proportion of people who have experienced VR, and its introduction into medicine should be relatively easy. To enable its use by a wide range of age groups, including older people, in the future it will be necessary to choose presentations based on age and individual preferences. The evolution of VR, augmented reality (AR), mixed reality (MR), and cross reality (XR) is proceeding apace, as is that of image technology^26^. If these issues can be resolved, VR has the potential to become a medical assistive device not only for resolving anxiety, but also for alleviating pain during dental treatment.

Our results showed that the use of VR during impacted mandibular third molar extraction under local anesthesia effectively alleviated fear and anxiety during treatment without side effects. They also suggested that pain relief may vary depending on the content of the presentation. VR has potential as a medical assistive device for use during outpatient surgery under local anesthesia.

## Subjects and methods

### Subjects

**Figure 3:**
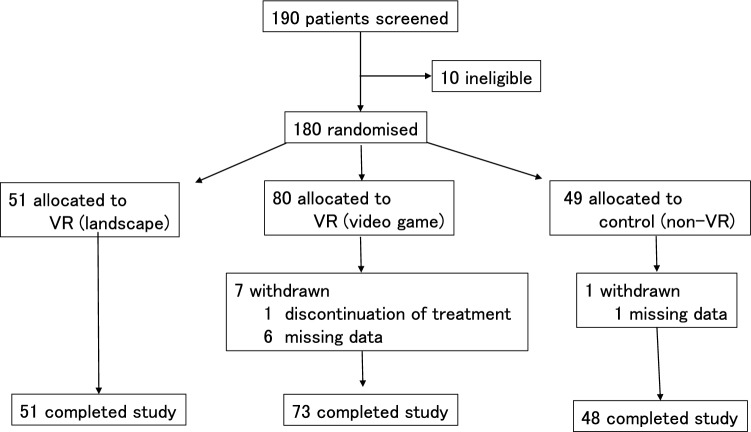
Subject selection. Ten of these patients were excluded because of a history of cybersickness or vertigo, pregnancy, or previous heart disease. The remaining 180 were randomly allocated to one of three groups: a non-VR group (n = 49), a natural landscape VR group (n = 51), and a video game VR group (n = 80). Data were collected from 172 of these patients (66 men and 106 women) who comprised the final study population.

The study population comprised patients who required impacted mandibular third molar extraction in the Department of Oral Surgery of Saga University Hospital between April 2020 and March 2021. For sample size calculation, we used G*Power software (ver. 3.1.9.7; Heinrich-Heine-Universität Düsseldorf, Düsseldorf, Germany), found that 100 participants (α level = 0.05, the power = 0.95, and the effect size = 0.5) with an actual target of at least 40 per group were suitable for statistical analysis, and a total of 190 participants were recruited. Ten of these patients were excluded because of a history of cybersickness or vertigo, pregnancy, or previous heart disease. The remaining 180 were randomly allocated to one of three groups by the envelope method: a non-VR group, a natural landscape VR group, and a video game VR group. Data were collected from 172 of these patients (66 men and 106 women) who comprised the final study population (Fig. 3). The demographics of the study population is shown in Table 1. The mandibular wisdom teeth were impacted horizontally, corresponding to Position A or B and class I or II in Pell and Gregory’s classification^27^. This study was approved by the Ethics Committee of the Faculty of Medicine, Saga University (approval number 2017-12-05) and was registered with UMIN Clinical Trials registry (UMIN000035594; 19/01/2019), and informed consent was obtained from all patients. The study complied with the Declaration of Helsinki, and the manuscript adheres to the applicable CONSORT guidelines.

### Evaluation method

As a subjective evaluation, preoperative and postoperative fear and anxiety were evaluated by a questionnaire that included a visual analog scale (VAS). Anxiety was scored on a scale from “Completely relaxed” at 0 mm to “The most anxious state imaginable” at 100 mm. The post-treatment questionnaire asked patients to evaluate their satisfaction, reduction in anxiety, and change in pain during treatment carried out while experiencing VR on a five-point scale (Table 5).

**Table 5:** Anxiety and pain questionnaire.

1. How did you feel measures while you performed surgical treatment with watching VR?
(Excellent · Good · Fair · Poor · Unacceptable)
2 How did your anxiety change by watching VR during surgical treatment?
(Greatly decreased · Decreased · Unchanged · Slightly increased · Increased)
3. How did your pain change by watching VR during surgical treatment?
(Greatly decreased · Decreased · Unchanged · Slightly increased · Increased)

### Surgical technique

After local infiltration anesthesia, the patients were placed in a supine position and were fitted with a patient monitor and a head-mounted display (Oculus Go 32 GB, Oculus VR, Menlo Park, CA). Before the impacted third molar extraction started, they experienced a VR presentation for a short period and were asked to confirm that they were not feeling uncomfortable or disturbed. The extractions were conducted by three board-certified oral surgeons. The following procedure is used for the extraction of an impacted third molar. First, a gingival incision is made for extraction. The subperiosteal mucoperiosteal flap is detached and the mandibular cortical bone is clarified. After removing the bone around the impacted wisdom tooth and clarifying the crown of the tooth, the crown and root of the tooth are divided with a cutting bar. After splitting, the crown and root are dislocated and removed respectively. After curettage of the extraction socket, the mucoperiosteal flap is restored to its original position and sutured to complete the procedure.

It was explained to the patients that should they feel uncomfortable or disturbed because of the VR during the extraction, the procedure would immediately be halted, and the VR removed. After the extraction was complete, the VR was removed, hemostasis was confirmed, and the procedure was concluded.

### VR presentations

**Figure 4:**
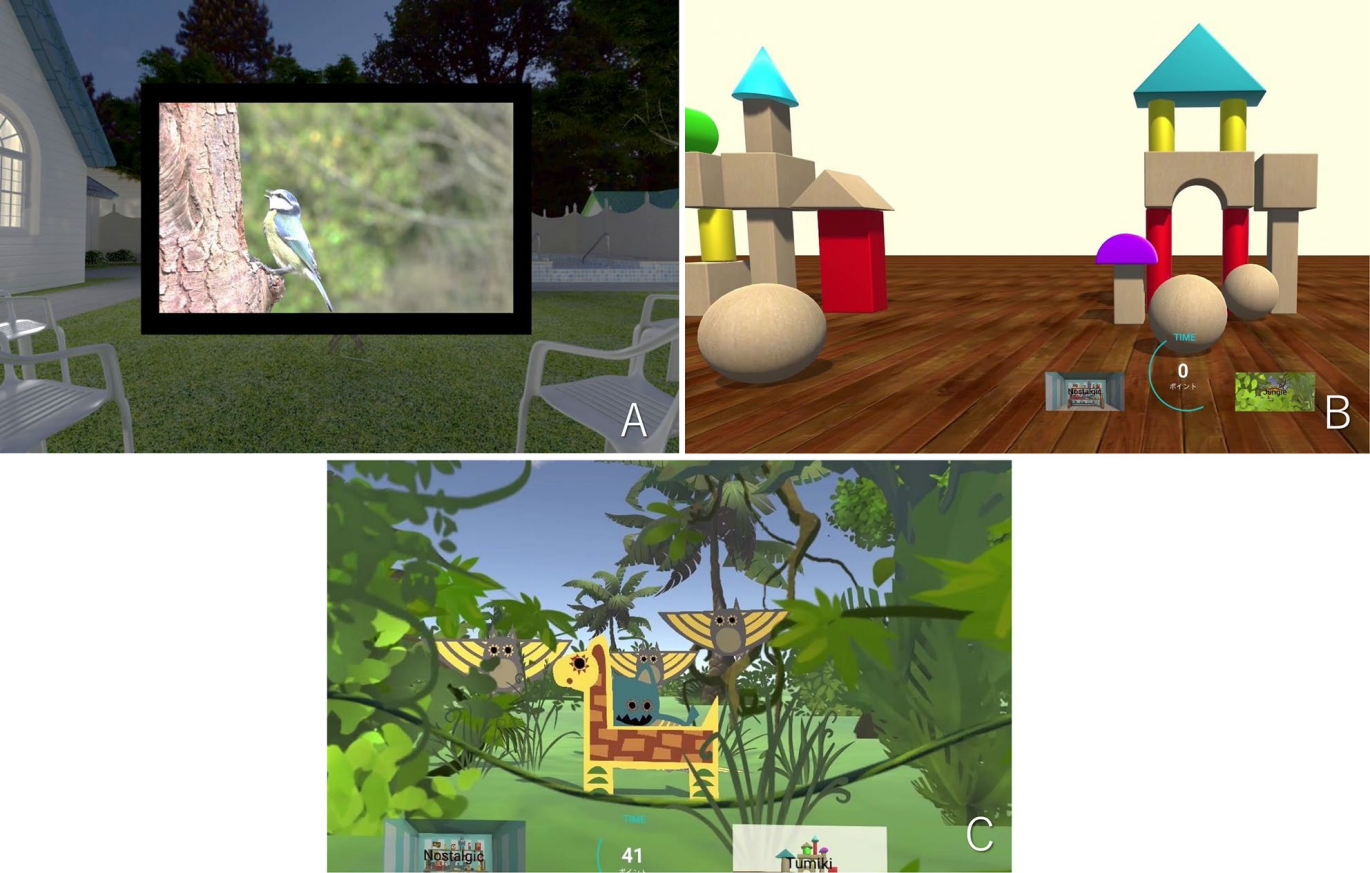
Prepared VR presentations. (**A**) Natural landscapes. An outdoor landscape was created on VR, with a large screen set up in the center on which images were displayed. The scenes shown on the screen were changed approximately every 3 min to avoid patients becoming bored with the images. (**B**) and (**C**): Video games. Games in which the viewer uses a controller to earn points by attacking targets. To prevent the subjects from becoming bored, they were permitted to choose freely among three different types of game during the procedure.

All prototype VR software we used for this study was specially developed by the company PR NETWORK Co. (Fukuoka, Japan). The author (YY) owns the copyright of this software. The natural landscape presentation was the presentation used in our previous study. Specifically, the VR reproduced a large film screen either in a cinema or set up outdoors (on a beach or in a garden), on which the images were projected. Natural scenes of animals exhibiting little movement, or of the sea or rivers, were projected onto the screen (Fig. 4A, Sup-1). We also produced a new, attack-type video game presentation. In this game, images of target-shooting and bowling were shown, with the user using a controller to attack the target and earn points (Fig. 4B, C, Sup-2). To prevent the subjects from becoming bored, they were permitted to choose freely among three different types of game during the procedure. To prevent them from shaking their heads during the presentation, the headset was set up so that the edge of the image disappeared if a subject moved their head so that they were no longer looking straight ahead. Music was matched to the images and played to the patients via the head-mounted display.

### Pain threshold measurement

This experiment was conducted on 46 healthy volunteers (9 men and 37 women; mean age 27.0 years). They experienced the VR presentations while lying flat in the same position as the patients who underwent the surgical procedure. Their pain thresholds were measured using a PainVision PS-2100 (Nipro Corporation, Osaka, Japan) to measure the level of electric current perceived as pain^28,29^. The probe that delivered the electric current of this machine was applied to the subject’s right forearm skin. Measurements were conducted to determine: (1) the minimal perceived current (without VR); (2) the current while experiencing the natural landscape VR; and (3) the current while experiencing the video game VR. Each of these measurements was conducted three times, and the mean values were calculated. Because the minimum perceived current varied widely between individuals, the rate of change for everyone was calculated.

### Analysis method

The data obtained were examined with respect to their variability and showed a normal distribution curve. A *t*-test was used for intergroup comparisons. As a test method, Tukey–Kramer’s HSD test was performed as a comparison of all three groups. All statistical analyses were performed using JMP Pro 12 (SAS Institute, Tokyo), a value of *P* < 0.05 (two sided) was considered statistically significant in all analyses.

### Supplementary Information

Supplementary Information 1.

Supplementary Video 1.

Supplementary Video 2.

Supplementary Information 2.

## Data Availability

All data generated or analysed during this study are included in this published article and its supplementary information files.
